# Clinical Outcomes in Children and Adolescents With Functional Neurological Disorders

**DOI:** 10.1192/bjo.2025.10116

**Published:** 2025-06-20

**Authors:** Raka Maitra, Isabella Conti, Alisha Shah, Lily Smythe, Benjamin Baig

**Affiliations:** ^1^South London and Maudsley NHS Trust, London, United Kingdom; ^2^Kings College London, London, United Kingdom

## Abstract

**Aims:** Functional Neurological Disorders (FND) are common but poorly understood causes of disabling neurological symptoms. Children with FND are likely to have more difficulties with education and mental health disorders and have experienced more adversity in childhood such as physical, emotional, and sexual abuse. Emergence of FND in childhood can predict long-standing difficulties into adulthood. Predictors of poor outcomes in adults include a long duration or very severe symptoms, comorbid depression, personality disorder, chronic pain, dysfunctional family dynamics, perceived stigma about FND and social isolation. Each of these represents potential therapeutic targets for children and adolescents with FND.

However, literature about FND in children is poor and few studies describe outcomes and optimal models of care. Here we aim to analyse outcomes of FND in children to look for clinical and demographic predictors of prognosis and potential treatment targets.

**Methods:** Through the South London and Maudsley NHS Foundation Trust (SLaM), Electronic Patient Journey System, an electronic case record search was completed and 196 patients were identified who had a diagnosis of ICD–10 Dissociative Disorders given before the age of 18 between 2015–2025. Of these patients, 133 have a Dissociate Disorder as their primary diagnosis. Individual case records were assessed for demographic and clinical information and outcomes were defined through case record descriptions.

**Results:** In the group of 133 patients, 68 (51.1%) were discharged with a favourable outcome, 13 (9.8%) were transferred to adult FND services, 5 (3.7%) were admitted as psychiatric inpatients, 11 (8.3%) discontinued treatment and outcome data is not available, 15 (11.3%) remain under the care of the specialist service for ongoing treatment. Twenty-four (18.3%) were seen for consultation but managed under non-SLaM services. Therefore, in cases with known outcomes, 68/109 (62%) were favourable. Predictors of transfer to adult FND services or psychiatric admission include comorbid self-harm and depression, duration of symptoms prior to referral to paediatric FND services and family unwillingness to accept the diagnosis of FND.

**Conclusion:** Evidence from this cohort would suggest that positive clinical outcomes can occur in a high number of cases. These patients appear to benefit from specialised services which may include psychoeducation, family and individual therapy and multidisciplinary working. Early diagnosis of FND and treatment of comorbid psychiatric issues may further enhance prognosis. Further controlled studies would highlight specific therapeutic targets and interventions for children with FND.

